# Both comorbidity and worse performance status are associated with poorer overall survival after external beam radiotherapy for prostate cancer

**DOI:** 10.1186/s12885-020-06812-6

**Published:** 2020-04-15

**Authors:** Miikka Lehtonen, Lauri Heiskanen, Petri Reinikainen, Pirkko-Liisa Kellokumpu-Lehtinen

**Affiliations:** 1 Faculty of Medicine and Life Sciences, University of Tampere, Tampere, Finland; 2 Department of Oncology, Tampere University Hospital, Tampere, Finland

## Abstract

**Background:**

In this retrospective study, we evaluated the biochemical recurrence rate, metastatic disease progression, and prostate cancer-specific and overall survival in patients curatively treated with external beam radiotherapy (EBRT) for early prostate cancer (PC). We also examined the prognostic effect of comorbidity by Charlson Comorbidity Index (CCI) and overall performance status by Eastern Clinical Oncology Group (ECOG) score.

**Methods:**

A total of 665 men treated between 2008 and 2013 were enrolled from Tampere University Hospital, Finland. Prostate-specific antigen (PSA) tests and hospital records were used to determine the 5-year survival for each aforementioned endpoint using a Kaplan-Meyer estimate. To analyze the impact of the selected prognostic factor, we used a Cox regression model to calculate the corresponding hazard ratio (HR) and 95% confidence interval (CI).

**Results:**

With a median follow-up-time of 7.12 years, the 5-year overall survival (OS) after EBRT was 88.9% [86.5 -91.3%], prostate cancer-specific survival (PCSS) was 97.9% [96.7 -99.1%], metastasis-free survival (MFS) 94.8% [93.0 -96.6%] and biochemical recurrence-free survival (BRFS) 88.7% [86.2 -91.2%]. Both CCI (HR = 1.38, [1.25–1.51]) and ECOG score (HR = 1.63, [1.29–2.05]) declined OS, as well as Gleason score and T score (*P* <  0.05). Gleason score and T grade also associated to worse PCSS, MFS and BRFS.

**Conclusions:**

CCI and ECOG score are useful tools in evaluating the overall life expectancy of the patient after EBRT for PC. T-stage and Gleason score remain still the major prognostic factors.

## Background

Prostate cancer (PC) is the most common cancer among men in developed countries worldwide. In Finland, 5162 new cases were reported in 2016 [1]. PC primarily affects older males, with a peak incidence in men over 65 years [2] and it accounted for 13.3% of all cancer-related deaths among men in 2016 [3]. With earlier diagnostics in the PSA (prostate-specific antigen) era and advancements in treatment options, the prognosis has steadily improved in the past 15 years. The most recent register data reported a 5-year survival rate as high as 93% in the entire country [4].

External beam radiotherapy (EBRT) is one of the most common treatments of early PC and is often combined with androgen deprivation therapy (ADT) for patients with intermediate and high-risk disease. For localized disease, radical prostatectomy is also a viable option, especially for younger patients with few comorbidities. Other treatment options include brachytherapy, active and passive surveillance and ADT [5, 6].

The present study aimed to evaluate the treatment outcomes of prostate cancer patients in Tampere University Hospital receiving EBRT as a curative treatment for localized PC and how comorbidity and overall fitness affect the results. We used Charlson Comorbidity Index (CCI) in measurement. CCI was developed in 1980’s and is eponymously named after its developer [7], and is still in common use. To measure overall performance in patients, we used Eastern Clinical Oncology Group (ECOG) score [8], which was also developed nearly 40 years ago and is equally still widespread.

Register data shows that the prostate cancer-specific survival (PCSS) rates of all patients treated in Tampere University hospital are among the best in Finland with 1-year and 5-year survival rates of 99 and 95%, respectively [4]. However, no previous study has exclusively evaluated the outcomes of patients treated with EBRT in this region.

## Methods

### Study population, data collection, treatment, and follow-up

The study population was comprised of PC patients enrolled in The Clinical Registry at the Department of Oncology in Tampere University Hospital between 2010 and 2013, as well as patient data retrieved from the hospital information system from 2008 and 2009. Patients were identified from the hospital information system with a specific code depicting EBRT for PC. All patients receiving EBRT as a first-line treatment with curative intent, regardless of tumor T-score and pre-existing risk factors, were included. Only patients who met the following criteria were excluded from this five-year patient population: 1) The EBRT ended after December 31, 2013; 2) The patient was not a resident of a municipality belonging to the Pirkanmaa Healthcare District (detailed follow-up data were unavailable); 3) Metastatic disease (M1); 4) Premature cessation of EBRT due to a sudden illness (unrelated to prostate cancer); 5) EBRT as a second-line treatment (failed androgen deprivation monotherapy or salvage radiation therapy after radical prostatectomy); and 6) No radical treatment (palliative radiotherapy).

**Fig. 1:**
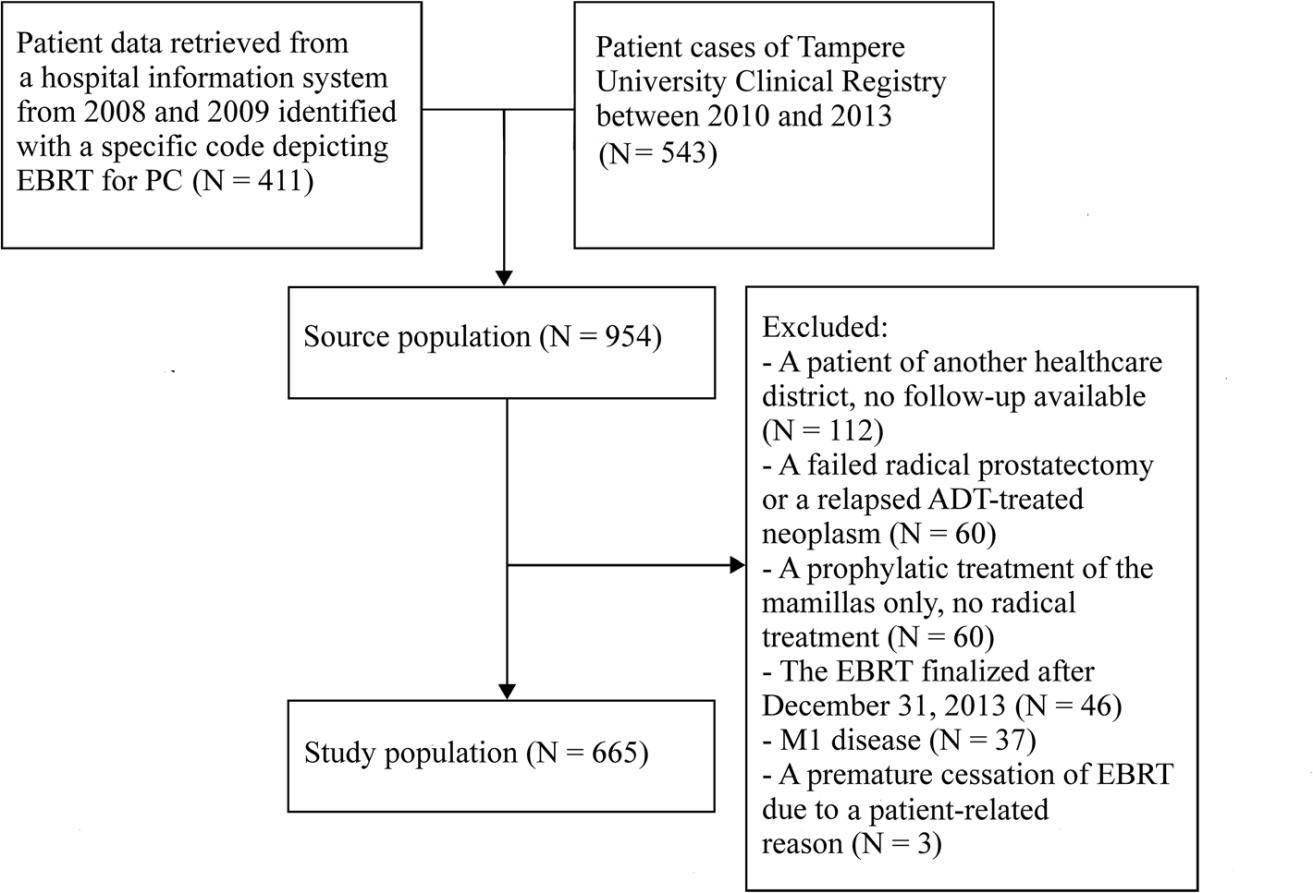
A model depicting the formation of the final study population. EBRT = External Beam Radiotherapy; PC = Prostate Cancer

The final population was comprised of 665 men (Fig. 1**)**. The study was approved by the ethical committee of the region, and permission to access patient report inquiries was granted by the director of the faculty of science (ETL R155025). The data collection occurred between May 2015 and March 2019 and included an assessment of the patient demographics, medical history and carcinoma-related details from the patient records of Tampere University and Tampere City Hospital.

Most men received treatment in the form of intensity-modulated radiation therapy (IMRT) with image-guided assistance (*N* = 646, 97.1%). The remaining cases were treated with either volumetric-modulated arc therapy (VMAT, *N* = 7, 1.1%) or three-dimensional conformal radiotherapy (3D-CRT, *N* = 12, 1.8%). Altogether, 367 men (55.1%) received androgen deprivation therapy (ADT) with a median duration of 20.3 months (range 1.6–127.4, N=). In 9 cases (2.5%), the duration of hormonal treatment could not be determined due to missing data. Among patients receiving ADT, 283 patients (76.9%) patients received a combined neoadjuvant-adjuvant –treatment, 74 patients (20.1%) received only the neoadjuvant and 11 patients (3.0%) only the adjuvant treatment.

Of patients belonging to a high recurrence risk group (*N* = 360) in the D’Amico classification [9], 295 men (81.9%) received ADT. In the intermediate-risk group (*N* = 183), 62 men (33.8%) received ADT. The median duration of the medicinal treatment in the high-risk group was 25.0 months (range [2.0–127.4], *N* = 288), and in the intermediate-risk group, it was 6.0 months (range [1.5–33.7], *N* = 61). In the low-risk group (*N* = 121), ADT was given to 10 men (8.3%). One patient could not be classified using the D’Amico system because of the inaccurate T grade documenting. A urologist decided to begin a neoadjuvant or adjuvant medication, based on the risk group and individual factors such as quality of life concerns. The patient had the right to decline from hormonal treatment. The long-term follow-up after EBRT was also mainly carried out by the department of urology and in lower-risk groups partly transferred back to primary healthcare.

ADT used most frequently was luteinizing-hormone-releasing hormone (LHRH) analog monotherapy with either leuprorelin or goserelin (*N* = 308, 83.9%). In 46 (12.5%) cases, this treatment was combined with antiandrogen bicalutamide. Two men (0.54%) received bicalutamide monotherapy, and 9 men (2.5%) received an LHRH-agonist (degarelix). Furthermore, two men (0.54%) participated in the SPCG-13 adjuvant phase III clinical trial and were treated with six cycles of docetaxel combined with a hormonal adjuvant treatment after radiotherapy [10].

The initial diagnosis was performed through a pathological examination of core needle biopsies of the prostate in a vast majority of the cases (*N* = 656, 98.6%). In nine cases (1.4%), cancer was an incidental finding after a routine examination of the surgical pathology slides after transurethral resection of the prostate (TURP). Standardly, a transrectal 12-core biopsy procedure was used, although there were patients with fewer or more biopsy cores (median 12.0, range [2−19], *N* = 612). The median percent of positive biopsy cores (PPC) was 40.0% (range 5.9% − 100%).

TNM-staging was established using both a pathology report and MRI imaging, through which the physician determined the clinical stage. Bone scans were performed to high-risk patients to exclude metastatic progression. The risk of lymph node and seminal vesicle metastasis was assessed by Memorial Sloan Kettering Cancer Center (MSKCC)-nomogram [11], and the radiation plan was selected accordingly. If the risk of seminal vesicle invasion was over 15% seminal apices were included in the treatment site and if lymph node involvement risk was over 35% pelvic lymph nodes were included in the radiation fields. Based on the nomogram, 452 men (67.9%) received treatment to the prostate gland and the bases of seminal vesicles alone. In 111 men (16.7%), seminal apices were included, and in 102 men (15.3%), both seminal apices and pelvic lymph nodes were radiated in addition to the prostate. Prostate and the bases of seminal vesicles were treated with 5 mm marginal. Treatment marginal to the seminal vesicle apices and lymph nodes was 7 mm. Most patients (*N* = 536, 80.6%) were treated with conventional fractionation (2 Gy, 5 times a week) with a dose of 78 Gy, which has been the standard of care until the recent introduction of hypofractionated schedules. A total of 32 men (4.8%) received hypofractionated radiotherapy treatment with fractions between 2.5–3.1 Gy. The detailed characteristics of the disease profiles and treatments are shown in Tables 1 and 2, respectively.

**Table 1:** Cancer and treatment characteristics of the study population

Characteristics	
Median age at the time of diagnosis (years; range)	70.9 (46.1–89.0)
T stage, n (%)	
T1	347 (52.2%)
T2a-b	62 (9.3%)
T2c	92 (13.8%)
T3	147 (22.1%)
T4	16 (2.4%)
unknown	1 (0.15%)
N1-disease, n (%)	5 (0.75%)
Gleason score, n (%)	
6	211 (31.7%)
7	260 (39.1%)
8	53 (8.0%)
9	138 (20.8%)
10	3 (0.45%)
Percentage of positive biopsy cores, n (%)	
1–10%	65 (9.8%)
11–20%	95 (14.3%)
21–30%	82 (12.3%)
31–40%	63 (9.5%)
41–50%	108 (16.2%)
51–60%	45 (6.8%)
61–70%	32 (4.8%)
71–80%	20 (3.0%)
81–90%	30 (4.5%)
91–100%	67 (10.1%)
Diagnostic transurethral resection of the prostate (TURP)	9 (1.4%)
Missing data	49 (7.4%)
Median PSA-level at the time of the diagnosis (range)	9.0 (0.9–694.0)
Median time from diagnosis to EBRT, months (range)	3.80 (0.77–83.6)
Median duration of ADT, months (range)	20.0 (1.6–125.7)
Fractionation type, n (%)	
conventional	633 (95.2%)
hypofractionated	32 (4.8%)
Average performance status *(ECOG score),* n (%)	
0	348 (52.3%)
1	281 (42.3%)
2	33 (5.0%)
3	3 (0.45%)
Charlson Comorbidity Index, n (%)	
0	298 (44.8%)
1	190 (28.6%)
2	98 (14.7%)
3	37 (5.6%)
4	20 (3.0%)
5	13 (2.0%)
6	6 (0.90%)
7	2 (0.30%)
8	1 (0.15%)

**Table 2:** Radiotherapy schedules of the study population

Characteristics	N	%
EBRT dose (Gy)		
60	3	0.45
62	7	1.1
66	1	0.15
67.5	1	0.15
70.2	20	3.0
72	61	9.2
74	27	4.1
75	1	0.15
76	4	0.60
78	536	80.6
80	4	0.60
Fraction size (Gy)		
2	633	95.2
2.5	1	0.15
2.6	1	0.15
2.7	20	3.0
3	3	0.45
3.1	7	1.1

Patient follow-up data were collected from the medical records of the urological or oncological departments at Tampere University Hospital and the urological department at the Tampere City Hospital. The PSA-levels were obtained from the Fimlab laboratory database used in every public health institution in Pirkanmaa Hospital District. Each patient attended a PSA laboratory control every 6 to 12 months and a doctor’s appointment at least once a year after the finalization of EBRT. If the patient had symptoms that could indicate a relapse, then the controls were taken more often. The dates of death were obtained from the Tampere University hospital patient records, which are directly synchronized with the Finnish Population Information System.

### Outcomes and statistical analysis

The endpoint for biochemical recurrence-free survival (BRFS) was defined as a PSA increase by 2.0 μg/l or more from the lowest accomplished value after EBRT (nadir). The endpoint for metastasis-free survival (MFS) was determined by metastatic lesions shown in imaging. The date of death was used to determine the endpoint for overall survival (OS) and prostate-cancer specific survival (PCSS). The cause of death was determined by examining the patient records before death or by an autopsy report in selected cases.

No routine CT-scans or plain X-rays were used in the follow-up, and patients were only imaged if they had symptoms that could indicate metastatic disease or if they experienced a biochemical failure. For patients who did not reach the primary endpoint, the last registered PSA-value, physical examination (physician’s appointment) or data collection date (whether the patient had died or not) was used to determine the follow-up time. Survival and follow-up times were determined from the date at which PC was diagnosed by a pathologist.

The data were analyzed using SPSS Statistics 23.0 (IBM Corporation, Armonk, NY, USA) statistical analysis software. By using the aforementioned endpoints, we plotted age-adjusted Kaplan-Meyer curves for BRFS, MFS, PCSS, and OS. To study potential prognostic factors, we used Cox proportional hazards regression model *(Forward: LR method)*. The factors included in the analysis were age at the time of diagnosis, Gleason score, PSA-level at diagnosis, T-stage, N-stage, ADT, ECOG-score and Charlson Comorbidity Index (CCI) score. The variables included in final models were chosen based on their significance preliminary models. *P*-values below 0.05 were considered statistically significant. The frequencies and weights of different Charlson comorbidities are shown in Table 3. CCI points are determined by summing the weights of the patient’s comorbidities.

**Table 3:** Patient comorbidities characteristics

Charlson Comorbidity	Weight	N	%
Diabetes without complications	1	129	19.4
Chronic pulmonary disease	1	94	14.1
Cerebrovascular disease	1	58	8.7
Myocardial infarction	1	57	8.6
Connective tissue disease	1	53	8.0
Congestive heart failure	1	30	4.5
Dementia	1	22	3.3
Peripheral vascular disease	1	18	2.7
Peptic ulcer disease	1	15	2.3
Liver disease, mild	1	2	0.30
Renal disease, moderate or severe	2	60	9.0
Diabetes with end organ damage	2	22	3.3
Malignant tumor (within five years)	2	17	2.6
Leukemia, polycythemia	2	4	0.60
Lymphoma, multiple myeloma	2	3	0.45
Hemiplegia	2	2	0.30
Liver disease, moderate or severe	3	2	0.30
Metastatic solid malignancy	6	0	0
Acquired immunodeficiency syndrome (AIDS)	6	0	0

To study the effects of performance status and comorbidity separately, we plotted two distinct models. In the first model, the CCI score was used as a categorical variant. Comorbidity was classified into three categories: no comorbidity (CCI = 0), mild to moderate comorbidity (CCI = 1–3) and severe comorbidity (CCI = 4 or more). In the second model, ECOG score was used as a categorical variant. Overall performance was classified: normal (ECOG = 0), mild restrictions (symptoms only during strenuous exercise, ECOG =1) and from moderate to severe restrictions (symptomatic during normal daily activities, ECOG = 2 or more). To assess the potential presence of multicollinearity in the models, we calculated variance inflation factors (VIFs). With all VIFs being under 1.4, no significant multicollinearity was found. A one-way ANOVA test was also performed.

## Results

In a median follow-up time of 7.12 years (standard deviation ±2.4 years, range 6.2–176.8 months), biochemical recurrence was observed in 137 (20.6%) patients. Among 367 men receiving ADT, 94 (25.6%) experienced a relapse, and for 24 of those (6.5%), the relapse occurred during the ongoing ADT treatment. The 5-year age-adjusted BRFS for the entire study population was 88.7% with a standard error (ste) of 0.013. The 95-% confidence interval (CI) was [86.2 -91.2%].

Altogether, 54 (8.1%) patients were diagnosed with metastatic disease during the follow-up. The 5-year MFS was 94.8% (ste: 0.009, [93.0 -96.6%]). The primary metastatic sites were bone (*N* = 43, 79.6%), lymph nodes (*N* = 17, 31.5%), lungs (*N* = 5, 9.3%), adrenal glands (*N* = 2, 3.7%), orbit (N = 1, 1.9%) and liver (N = 1, 1.9%).

158 men (23.8%) died during the follow-up. The 5-year age-adjusted PCSS was 97.9% (ste: 0.006, [96.7 -99.1%]), and the 5-year OS was 88.9% (ste: 0.012, [86.5 -91.3%]). Three leading causes of death were cardiovascular disease (*N* = 39, 24.7%), followed by other malignancies than prostate cancer (*N* = 33, 20.9%) and finally prostate cancer (*N* = 31, 19.6%). The cause of death remained unknown in 13 cases (8.2%) but was unlikely prostate cancer-related, as no biochemical recurrence or metastatic disease was registered for these cases. Other causes included neurological (including dementia, *N* = 18, 11.4%), infection (*N* = 10, 6.3%), pulmonary fibrosis or COPD (*N* = 9, 5.7%), trauma (N = 3, 1.9%) and uremia (*N* = 2, 1.3%).

### Prognostic factors

**Fig. 2:**
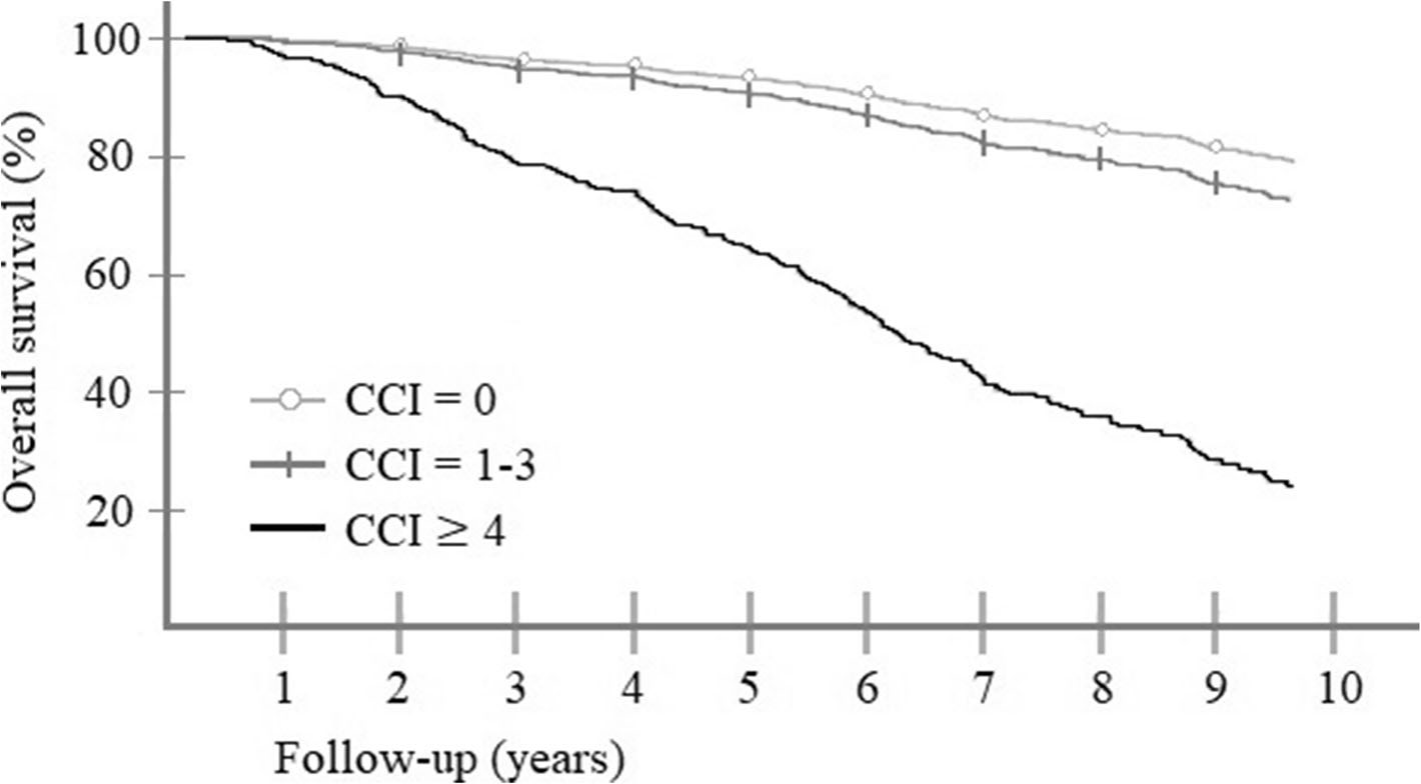
Cox proportional hazards model of overall survival after EBRT in different Charlson Comorbidity groups. Otherwise healthy men are the baseline, CCI ≥ 4 are severely comorbid, and CCI = 1–3 are men with mild to moderate comorbidity. CCI = Charlson Comorbidity Index

The main findings considering prognostic factors on overall survival are listed in Table 4***.*** In the first model, we evaluated how Charlson Comorbidity Index influenced overall survival after EBRT (Fig. 2). Overall, CCI had a statistically significant effect (P *= < 0.001*). Compared to the baseline patients with no comorbidity (CCI = 0*, N = 298*), the population with severe comorbidity (CCI = 4, *N = 42*) had over 6-fold increased a risk of death with a hazard ratio (HR) of 6.11 (95-% CI = [3.76–9.92], P = < 0.001). Men with mild to moderate comorbidity (CCI = 1–3, *N = 324*), had not a statistically significant difference compared to the CCI = 0 population (HR = 1.38, [0.97–1.97], *P* = 0.078). Other factors that had an effect on overall survival were Gleason score (HR = 1.21, [1.04–1.41], *P* = 0.015), T-stage (HR = 1.11, [1.01–1.21], *P* = 0.030) and overall performance score (HR = 1.63, [1.29–2.05], P = < 0.001). Androgen deprivation therapy (*P* = 0.70), age (*P* = 0.27), N-grade (*P* = 0.75) and PSA-value before diagnosis (*P* = 0.15) were not a statistically significant prognostic factors in these patients.

**Table 4:** Prognostic factors associated with overall mortality after EBRT

***Model 1.****Charlson Comorbidity Index used as categorical variant.*			
**Factor**	**HR**	**95-% CI**	***P*-value**
CCI = 0 *(N = 298)*			< 0.001
CCI = 1–3 *(N = 324)*	1.38	[0.97–1.97]	0.078 ***(NS)***
CCI = 4 or more *(N = 42)*	6.11	[3.76–9.92]	< 0.001
Gleason score	1.21	[1.04–1.41]	0.015
T-grade	1.11	[1.01–1.21]	0.030
Zubrod score	1.63	[1.29–2.05]	< 0.001
*Not significant:* Androgen deprivation therapy (P = 0.70), age (P = 0.27), N-grade (P = 0.75), PSA-value before diagnosis (P = 0.15).			
***Model 2.****Performance status used as categorical variant.*			
Z = 0 *(N = 348)*			< 0.001
Z = 1 *(N = 281)*	2.20	[1.54–3.13]	< 0.001
Z = 2 or more *(N = 36)*	2.22	[1.21–4.09]	0.010
Charlson Comorbidity Index	1.38	[1.25–1.51]	< 0.001
Gleason score	1.19	[1.02–1.39]	0.026
T-grade	1.11	[1.02–1.22]	0.022

*Not significant:* Androgen deprivation therapy (*P* = 0.88), age (*P* = 0.18), N-grade (*P* = 0.77), PSA-value before diagnosis (*P* = 0.080)

*N* = 665. Abbreviations: *NS* not significant, *HR* hazard ratio, *CI* confidence interval

**Fig. 3:**
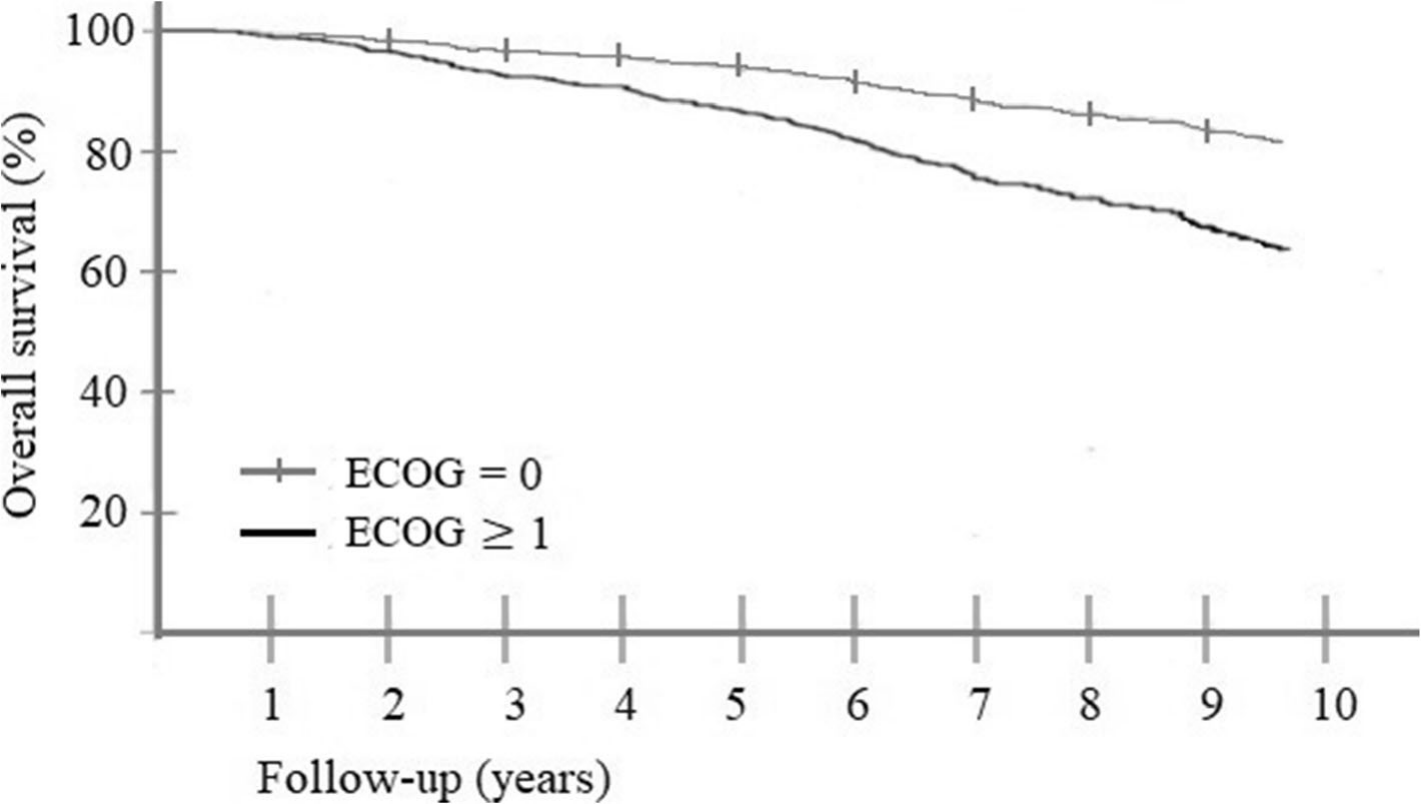
Cox proportional hazards model of overall survival in different ECOG score groups. ECOG = 0 have no disability, ECOG ≥1 have mild to severe disability. ECOG = Eastern Clinical Oncology Group score

In the second model, overall performance score was used as categorical variant (Fig. 3). Compared to the baseline (ECOG = 0, *N = 348*), men with mild restrictions (Z = 1, *N = 281*) had an increased risk of death (HR = 2.20, [1.54–3.13], P = < 0.001). Similarly, men with moderate to severe restrictions (ECOG ≥2, *N = 36*) had an increased risk (HR = 2.22, [1.21–4.09, *P* = 0.010) compared to the ECOG = 0 patients. There was not a statistically significant difference between groups ECOG = 1 and ECOG ≥2. Other factors that increased the risk (as in Model 1) were Gleason score (HR = 1.19, [1.02–1.39, *P* = 0.026) and T-stage (HR = 1.11, [1.02–1.22], *P* = 0.022), as well as CCI score (HR = 1.38, [1.25–1.51], P = < 0.001).

Neither comorbidity nor overall performance score increased the risk of biochemical recurrence (*P*-values 0.24 and 0.15, respectively), emergence of the first metastasis (P-values 0.59 and 0.83) or prostate-cancer related mortality (P-values 0.076 and 0.31). T-stage (HR = 1.23, [1.11–1.36], *P* <  0.001) and Gleason score (HR = 1.19, [1.02–1.41], *P* = 0.036) increased the risk of biochemical relapse. T-stage (HR = 1.29, [1.08–1.53], *P* = 0.004), N-stage (HR = 4.01, [1.22–13.1], P = 0.022) and Gleason score (HR = 1.63, [1.24–2.15], P <  0.001) declined the metastasis-free survival. T-stage (HR = 1.52, [1.19–1.94], *P* = 0.001) and Gleason score (HR = 1.44, [1.01–2.06], *P* = 0.044) increased the risk of prostate-cancer death. In sub-group analysis, whether the patient was hypofractionated or not, had not any effect on OS, PCSS, MFS or BRFS (*P* > 0.9).

## Discussion

Our results show that the radical radiotherapy treatment results of early prostate cancer are excellent. Overall the 5-year OS (88.9%), PCSS (97.9%), MFS (94.8%) and BRFS (88.7%) were similar or better compared with the figures reported in other studies [12–18]. In recent years, there have been some large high-quality population-based studies that have demonstrated an association between increased overall mortality and comorbidity [19–21]. Smaller studies have found similar results earlier [22–24]. CCI has been shown to be a continuous variable in larger studies [19, 20], and we would probably have noticed a statistically significant effect with greater *N* in group CCI = 1–3.

Radiotherapy remains still a very important curative treatment of early prostate cancer with or without ADT. ADT increases the risk of myocardial infarction and diabetes, but the absolute risk increases similarly whether the patient has pre-existing conditions or not according to previous studies [25]. Adjuvant chemotherapy with docetaxel did not improve biochemical disease-free survival after radical RT according to the recent results of Scandinavian Prostate Cancer Group trial-13 (SPCG-13) [10]. Based on our results we should more carefully take into account patients’ comorbidities and performance status when selecting treatment options for the elderly patient population.

Compared to earlier studies, this study showed that comorbidity and overall performance score affect overall survival independently. Most previous studies have focused on the Charlson Comorbidity Index alone. This study also used a differed stratification compared to previous studies. Both Rajan and Berglund used CCI ≥ 3 as a threshold for severe comorbidity [19, 20], but we demonstrated with a quite small *N* = 62 that in group CCI ≥ 4 patients have a 3.8–10 times the risk of dying after EBRT compared to healthy. CCI ≥ 4 could be a threshold value if the Charlson Comorbidity Index is used in daily practice in deciding the suitable treatment.

The present study had several limitations. This was an observational retrospective study without randomization or blinding. The number was quite small and comprised of 665 men. However, all the patients were treated in the same institution according to the same guidelines. Additional strengths of this study include very careful data collecting and non-selectiveness. We did not exclude any patients due to age, general condition or functioning-related factors, and the present cohort is hence comparable to the actual patient population treated with radiation therapy in general hospitals. The analysis of the material was quite comprehensive. However, we did not collect data on all possible contributing factors, such as familial history of prostate cancer or marital status. Some additional factors, such as the percentage of cancer volume (PCV), were investigated in preliminary models but then dropped due to lacking significance compared to other factors. We focused on survival and did not address matters such as quality of life or adverse effects of the treatment, which could be important from patient’s perspective.

## Conclusion

Charlson comorbidity is associated with weaker overall survival after EBRT for prostate cancer even if the overall performance status of the patient is considered, and both CCI and ECOG score have an independent effect. More study is needed, at which point exactly patient’s disease burden and overall fitness should exclude EBRT.

## Data Availability

The datasets used and/or analyzed during the current study are available from the corresponding author on reasonable request.

## Abbreviations

3D-CRT: Three-dimensional Conformal Radiotherapy
ADT: Androgen Deprivation Therapy
ANOVA: Analysis of Variance
BRFS: Biochemical recurrence-free Survival
CI: Confidence Interval
CCI: Charlson Comorbidity Index
COPD: Chronic Obstructive Pulmonary Disease
CT: Computer Tomography
EBRT: External Beam Radiotherapy
ECOG: Eastern Clinical Oncology Group
Gy: Gray
HR: Hazard Ratio
IMRT: Intensity-modulate Radiation Therapy
LHRH: Luteinizing-hormone-releasing Hormone
MFS: Metastasis-free Survival
MRI: Magnetic Resonance Imaging
MSKCC: Memorial Sloan Kettering Cancer Center
OS: Overall Survival
PC: Prostate Cancer
PCSS: Prostate Cancer-specific Survival
PCV: Percentage of Cancer Volume
PPC: Percentage of Positive Biopsy Cores
PSA: Prostate-specific Antigen
SPCG: Scandinavian Prostate Cancer Group
TURP: Transurethral Resection of the Prostate
VIF: Variance Inflation Factor
VMAT: Volumetric-modulated Arc Therapy
